# Different HLA-DRB1 allele distributions in distinct clinical subgroups of sarcoidosis patients

**DOI:** 10.1186/1465-9921-11-25

**Published:** 2010-02-26

**Authors:** Johan Grunewald, Boel Brynedal, Pernilla Darlington, Magnus Nisell, Kerstin Cederlund, Jan Hillert, Anders Eklund

**Affiliations:** 1 Department of Medicine, Division of Respiratory Medicine, Karolinska University Hospital Solna, Sweden; 2 Department of Clinical Neuroscience, Division of Neurology, Karolinska University Hospital Solna, Sweden; 3 Department of Medicine, Södersjukhuset (Stockholm South General Hospital), Sweden; 4 Department of Clinical Science, Intervention and Technology (CLINTEC), Division of Radiology, Karolinska University Hospital Huddinge, Sweden; 5 All from Karolinska Institutet, Stockholm, Sweden

## Abstract

**Background:**

A strong genetic influence by the MHC class II region has been reported in sarcoidosis, however in many studies with different results. This may possibly be caused by actual differences between distinct ethnic groups, too small sample sizes, or because of lack of accurate clinical subgrouping.

**Subjects and methods:**

In this study we HLA typed a large patient population (n = 754) recruited from one single centre. Patients were sub-grouped into those with Löfgren's syndrome (LS) (n = 302) and those without (non-Löfgren's) (n = 452), and the majority of them were clinically classified into those with recovery within two years (resolving) and those with signs of disease for more than two years (non-resolving). PCR was used for determination of HLA-DRB1 alleles. Swedish healthy blood donors (n = 1366) served as controls.

**Results:**

There was a dramatic difference in the distribution of HLA alleles in LS compared to non-LS patients (p = 4 × 10^-36^). Most notably, DRB1*01, DRB1*03 and DRB1*14, clearly differed in LS and non-LS patients. In relation to disease course, DRB1*07, DRB1*14 and DRB1*15 generally associated with, while DRB1*01 and DRB1*03 protected against, a non-resolving disease. Interestingly, the clinical influence of DRB1*03 (good prognosis) dominated over that of DRB1*15 (bad prognosis).

**Conclusions:**

We found several significant differences between LS and non-LS patients and we therefore suggest that genetic association studies in sarcoidosis should include a careful clinical characterisation and sub-grouping of patients, in order to reveal true genetic associations. This may be particularly accurate to do in the heterogeneous non-LS group of patients.

## Background

Sarcoidosis is an inflammatory disease of unknown aetiology. A genetic influence has been suggested by reports showing different susceptibilities and clinical manifestations between distinct ethnic groups [1]. In addition genetic linkage analyses have pointed out a significant linkage for the major histocompatibility complex (MHC) region [2], but also with minor peaks in specific regions of some other chromosomes [3]. One recently published genome wide association study described an association with the gene coding for Annexin A11, a protein with several important functions, including for apoptosis [4].

Sarcoidosis is thus influenced by a multitude of genes, however with the strongest associations described within the HLA-region. Several specific HLA alleles clearly associate with disease risk, and in a few cases also associations with disease phenotype e.g. uveitis [5] have been described. The HLA-DRB1*03/DQB1*02 haplotype has repeatedly been associated with Löfgren's syndrome [6-12] i.e. a distinct form of sarcoidosis characterised by an acute onset with bilateral hilar lymphadenopathy (BHL) and in some cases parenchymal infiltrates, erythema nodosum (EN) and/or bilateral ankle arthritis or distinct periarticular inflammation, and usually fever [13]. Our own group recently reported a strong influence by the DRB1*03 allele on the disease course in patients with Löfgren's syndrome, with complete recovery within two years in almost all DRB1*03 positive patients, but only in about half of the DRB1*03 negative patients [14]. Patients with Löfgren's syndrome may in fact constitute a disease entity by its own, and a recent report on specific associations between a CCR2 haplotype and Löfgren's syndrome support this hypothesis [15].

However, because of genetic differences, it may be difficult to compare the importance of specific HLA-DR alleles for the disease between various ethnic groups. The HLA-DRB1*03 allele is for example rather common in e.g. Scandinavia but extremely uncommon in Japanese individuals, so that the common association in Caucasians between DRB1*03 and an acute disease is rare in Japanese [16]. Most previous studies did not report distinct clinical sub-grouping of patients, but for example in our region, patients with Löfgren's syndrome make up 35-40% of all our patients and they will therefore contribute substantially to any genetic association. In the present study we therefore sub-grouped our patients into those with Löfgren's syndrome (LS), and those without Löfgren's syndrome (non-LS), and analysed any correlations between distinct HLA-DRB1 alleles and the risk for disease as well as the disease course. We studied a large group of sarcoidosis patients from one single centre (Karolinska University Hospital in Stockholm, Sweden).

## Materials and methods

### Study subjects

Altogether 754 patients were included in this study, 452 classified as non-Löfgren's syndrome (non-LS) and 302 as Löfgren's syndrome (LS) patients (se Table 1 for demographics).

**Table 1:** Patients' and controls'demographics

	All patients	Non Löfgren	Löfgren's syndrome
Female/male	330/424	194/258	136/166
*Age (years)	38 (19-77)	40 (19-77)	36 (23-71)
**Radiografic stage 0/I/II/III/IV	39/368/241/84/22	38/135/173/84/22	1/233/68/0/0
Never smokers	447	250	197
***Ex-smokers	216	154	62
Current smokers	90	48	42
****Treatment/no treatment	309/445	243/209	66/236

*values show median (min - max)

**one LS patient had BHL on disease onset but at the time for bronchoscopy and BAL the radiographic picture was classified as normal

***complete smoke stop since at least one year before analysis

****show number of patients on treatment with oral steroids or other immunosuppressives, including patients where treatment was initiated after bronchoscopy

All patients were referred to us for making the primary diagnosis through our examinations and investigations, and further management according to our routines at the outpatient clinic at the Pulmonary Division at the Karolinska University Hospital, Solna, Sweden. The vast majority of patients was seen by one of the authors (A.E), essentially from 1994 and onwards, and only in a few cases data was collected through records. Chest radiographies were evaluated by one of the authors (K.C), who is subspecialized in chest radiology with long experience of interstitial pulmonary disease. Patients were diagnosed with sarcoidosis through typical clinical manifestations, findings at bronchoscopy with bronchoalveolar lavage (BAL) including an elevated CD4/CD8 BAL cell ratio, and positive biopsies, using the criteria as outlined by the World Association of Sarcoidosis and other Granulomatous disorders (WASOG) [17].

Through the clinical characterisation, patients were grouped into those with Löfgren's syndrome, i.e. with an acute onset with bilateral hilar lymphadenopathy (BHL) and in some cases parenchymal infiltrates, erythema nodosum (EN) and/or bilateral ankle arthritis or distinct periarticular inflammation, and usually fever (n = 302) [18]. The remaining group of patients were classified as "non-Löfgren's" (n = 452). Moreover, 724 of the patients were followed for at least two years and grouped into those with a resolving or a non-resolving disease. A resolved disease was classified in patients who had normalised radiography and pulmonary lung function, no symptoms, and normal biochemical parameters. A non-resolved disease was defined in patients with signs of persistence or progress of chest radiographic changes or of deteriorating pulmonary function tests, with clinical signs of disease activity with symptoms (usually extreme fatigue, cough, dyspnea, or in patients who developed symptoms from extrathoracic organs that were caused by sarcoidosis). Thus a number of parameters were evaluated in order to correctly classify the patients. The non-resolving group may include patients with a slowly resolving, a chronic stable, or a chronic progressive disease. This whole group is of great importance to include and further sub-group in future studies.

The control group consisted of 1.366 consecutively collected Scandinavian blood donors, 815 female and 551 males, all residents of the Stockholm area.

All included subjects gave their informed consent for participation in the study, which was approved by the local ethics committee at the Karolinska University Hospital.

### Preparation of DNA and HLA typing

All patients were HLA-typed. Genomic DNA was extracted from whole blood samples, and HLA-DRB1 alleles were determined in every patient using the PCR-SSP technique [19]

### Statistical analyses

The effect of carriage of each allel was assessed by logistic regression in each subpopulation. DRB1*13 was used as a baseline comparator since this allele had approximately the same frequency in all subpopulations of cases and controls (see Table 1). The least common alleles were joined into an X term, thus including DRB1*09, DRB1*10, DRB1*12 and DRB1*16. It should be noted that odds ratios obtained in a logistic regression framework are adjusted for all the other alleles included in the model. Correction for multiple testing was performed for all tests simulaneously using false discovery rate (FDR) restriction, where a FDR of less than 5% was considered acceptable [20].

The difference in distribution of carriage of alleles in different sub-groups was assessed with a Χ^2 ^test. The non-parametric Mann Whitney test was used to compare age in the patient subgroups.

## Results

The HLA-DRB1 allele distribution frequencies were found to be significantly different in patients with LS versus non-LS patients (p = 4 × 10^-36^). We therefore analysed the DRB1 allele distibution in the entire group of sarcoidosis patients but also separately in LS and non-LS patients. In each of these patient groups, we moreover classified patients as resolving, with no signs of disease at two years after disease onset, or as non-resolving, with signs of disease at two years. Our data was compared with a large group of Swedish healthy controls (n = 1366).

The allele carrier frequencies for all patients and controls are shown in Table 2. As can be seen, the frequency of DRB1*13 was quite constant across all patient groups, and it was therefore chosen as a baseline comparator. Most of our significant findings are from DRB1 alleles that are frequently occurring. Thus DRB1*01, DRB1*03 and DRB1*15 are normally found in 22, 24 and 30% of healthy controls, respectively, and the substantial different frequencies in the patient groups will therefore be of importance for large numbers of individuals.

**Table 2:** Allele carrier frequencies (%) in controls (n = 1366), in all patients (n = 754), in non-Löfgren's patients (n = 452) and in Löfgren's syndrome patients (n = 302).

DRB1														
	*01	*03	*04	*07	*08	*09	*10	*11	*12	*13	*14	*15	*16	**x**

**Controls**	22.3	23.8	34.3	15.4	10.5	2.9	1.8	11.4	3.8	26.7	4.6	29.7	1.3	9.7
														
**All patients**	13.9	37.5	27.3	11.7	8.1	2.7	1.7	13.0	4.6	25.6	7.4	34.6	0.7	9.7
*Non-resolving	10.5	12.0	29.3	15.8	11.0	3.3	2.3	15.8	6.0	25.8	10.3	43.1	0.8	12.3
***Resolving***	16.9	66.2	25.5	6.5	5.2	2.2	1.2	10.5	2.8	25.2	4.6	24.9	0.3	6.5
														
**Non-Löfgrens**	10.2	16.8	30.8	14.4	10.8	3.1	2.0	17.0	5.5	26.3	9.1	39.4	0.7	11.3
***Non-resolving***	9.0	13.0	28.5	16.4	11.6	3.1	2.3	17.2	6.5	25.7	10.2	41.0	0.8	12.7
***Resolving***	12.0	30.4	38.0	7.6	8.7	3.3	1.1	17.4	2.2	29.4	5.4	33.7	0.0	6.5
														
**Löfgrens**	19.5	68.5	22.2	7.6	4.0	2.0	1.3	7.0	3.3	24.5	5.0	27.5	0.7	7.3
***Non-resolving***	22.2	4.4	35.6	11.1	6.7	4.4	2.2	4.4	2.2	26.7	11.1	60.0	0.0	8.9
***Resolving***	18.9	80.3	20.6	6.0	3.9	1.7	1.3	7.7	3.0	23.6	4.3	21.5	0.4	6.4

X = the least common alleles joined (DRB1*09, DRB1*10, DRB1*12 and DRB1*16)

*Within each patient group, allele frequencies for those with a non-resolving disease and those with a resolving disease are shown as well.

### All patients (n = 754)

In the complete group of patients, the strongest association was found for HLA-DRB1*03, with an OR of 1.91, p = 1 × 10^-6^. Also DRB1*14 (OR 1.79, p = 0.017) associated with disease, with a similar tendency for DRB1*15 (OR 1.32, p = 0.067). In contrast, DRB1*01 protected against disease (OR 0.61, p = 0.003), with a similar trend for DRB1*04 (OR 0.77, p = 0.086) (Table 3).

**Table 3:** Odds ratios (OR) of HLA-DRB1 alleles in all patients with sarcoidosis as compared to healthy Swedish controls (n = 1366).

	*All patients (n = 754)		Non-resolving patients (n = 399)		Resolving patients (n = 325)	
***DRB1 allele***	***OR***	***p-value***	***OR***	***p-value***	***OR***	***p-value***

*01	0.61	0.003	0.41	3 × 10^-5^	0.87	0.665
*03	1.91	1 × 10^-6^	0.45	1 × 10^-4^	5.42	3 × 10^-22^
*04	0.77	0.086	0.77	0.168	0.78	0.288
*07	0.77	0.165	0.93	0.844	0.44	0.009
*08	0.80	0.353	0.95	0.891	0.60	0.168
*11	1.21	0.368	1.41	0.136	0.98	0.98
*14	1.79	0.017	2.14	0.005	1.20	0.759
*15	1.32	0.067	1.55	0.011	1.01	0.985
x	1.09	0.775	1.22	0.484	0.79	0.573

*Separate figures are shown for all patients, non-resoving patients, and resolving patients.

X = the least common alleles joined (DRB1*09, DRB1*10, DRB1*12 and DRB1*16)

The distribution of alleles between resolving and non resolving disease was significantly different (p = 3 × 10^-39^), and these two groups were thus analysed separately. Among non-resolving patients (n = 399), the strongest associations were found for DRB1*01 and DRB1*03, both clearly protecting against a non-resolving disease (OR 0.41, p = 3 × 10^-5 ^and OR 0.45, p = 1 × 10^-4 ^respectively). Strong positive associations were here found for DRB1*14 (OR 2.14, p = 0.005) and DRB1*15 (OR 1.55, p = 0.011).

Among patients with a resolving disease, we noted an extremely strong positive association with DRB1*03 (OR 5.42, p = 3 × 10^-22^), in line with a recent study of ours [14]. In contrast, we found DRB1*07 to prevent a resolving disease among all patients (OR 0.44, p = 0.009).

### Non-Löfgren's patients (n = 452)

In this group of patients, HLA-DRB1*14 (OR 1.87, p = 0.021) associated with disease, with similar trends for DRB1*11 (OR 1.48, p = 0.067) and DRB1*15 (OR 1.35, p = 0.086) (Table 4). In contrast, HLA-DRB1*01 (OR 0.39, p = 5 × 10^-6^) and DRB1*03 (OR 0.63, p = 0.017) protected against disease (Table 3). In particular, HLA-DRB1*01 (OR 0.34, p = 5 × 10^-6^) and HLA-DRB1*03 (OR 0.47, p = 6 × 10^-4^) protected against a non-resolving disease, encompassing 78% of the non-Löfgrens patients. HLA-DRB1*14 (OR 2.08, p = 0.010) was instead a risk factor for non-resolving disease, with a similar tendency for HLA-DRB1*11 (OR 1.51, p = 0.085) and HLA-DRB1*15 (OR 1.42, p = 0.067). These stratified groups were significantly different with regards to allele distribution (p = 0.002).

**Table 4:** Odds ratios (OR) of HLA-DRB1 alleles in patients with non-Löfgren's sarcoidosis as compared to healthy Swedish controls (n = 1366).

	All non-Löfgren's patients (n = 452)		Non-resolving non-Löfgren's patients (n = 354)		Resolving non-Löfgren's patients (n = 92)	
***DRB1 allele***	***OR***	***p-value***	***OR***	***p-value***	***OR***	***p-value***

*01	0.39	5 × 10^-6^	0.34	5 × 10^-6^	0.48	0.107
*03	0.63	0.017	0.47	6 × 10^-4^	1.23	0.649
*04	0.80	0.238	0.73	0.110	1.04	0.947
*07	0.85	0.489	0.95	0.891	0.44	0.122
*08	0.93	0.852	0.97	0.947	0.79	0.755
*11	1.48	0.067	1.51	0.085	1.51	0.350
*14	1.87	0.021	2.08	0.010	1.13	0.898
*15	1.35	0.086	1.42	0.067	1.10	0.855
x	1.12	0.755	1.26	0.431	0.62	0.467

*Separate figures are shown for all patients, non-resoving patients, and resolving patients.

X = the least common alleles joined (DRB1*09, DRB1*10, DRB1*12 and DRB1*16)

### Löfgren's syndrome patients (n = 302)

HLA-DRB1*03 strongly associated with Löfgren's syndrome (OR 6.71, p = 2 × 10^-24^), and especially with a resolving form of the disease (OR 11.42, p = 8 × 10^-27^) (Table 5). DRB1*03 also tended to protect against a non-resolving form of Löfgren's syndrome (OR 0.20, p = 0.086). DRB1*15 was a risk factor for non-resolving disease in Löfgren's syndrome patients (OR 3.02, p = 0.016). In analogy with these distinct associations, the alleles distribution in the two stratified groups was significantly different (p = 3.8 × 10^-10^).

**Table 5:** Odds ratios (OR) of HLA-DRB1 alleles in patients with Löfgren's syndrome as compared to healthy Swedish controls (n = 1366).

	All Löfgren's syndrome patients (n = 302)		Non-resolving Löfgren's syndrome patients (n = 45)		Resolving Löfgren's syndrome patients (n = 233)	
***DRB1 allele***	***OR***	***p-value***	***OR***	***p-value***	***OR***	***p-value***

*01	1.12	0.755	1.08	0.929	1.16	0.730
*03	6.71	2 × 10^-24^	0.20	0.086	11.42	8 × 10^-27^
*04	0.73	0.190	1.12	0.882	0.68	0.165
*07	0.60	0.116	0.80	0.831	0.47	0.067
*08	0.50	0.104	0.71	0.769	0.52	0.183
*11	0.66	0.246	0.47	0.487	0.72	0.443
*14	1.44	0.443	2.87	0.129	1.24	0.775
*15	1.32	0.264	3.02	0.016	1.00	0.995
x	0.96	0.947	1.00	0.999	0.89	0.855

*Separate figures are shown for all patients, non-resoving patients, and resolving patients.

X = the least common alleles joined (DRB1*09, DRB1*10, DRB1*12 and DRB1*16)

## Discussion

In contrast to most previous studies and in order to better reveal genetic associations with sarcoidosis, we here sub-grouped patients into those with LS, with distinct clinical manifestations, and those without this syndrome. We found the allele carrier frequencies in these two patient sub-groups to clearly be quite different. Future studies on gene associations in sarcoidosis should accordingly take into account the number of LS patients in the study group, and preferably investigate the LS patient group separately. All patients in the present study represent a large homogeneous population of sarcoidosis patients derived from one single centre in Stockholm, Sweden. The vast majority of cases were seen and clinically evaluated by one of the authors. One important observation in the present study is that more than half of all patients developed a non-resolving disease. In this group of patients, many will develop a chronic inflammatory disease with sometimes lifelong need of attention of the health care system. Although we cannot exclude the possibility that the patient cohort from our clinical centre is somewhat biased and may include unusually large numbers of patients with a more severe disease, in this study population sarcoidosis seems to be a disease with more profound negative health effects than previously thought. In other ethnic groups this may be even more pronounced.

In the entire patient group, especially DRB1*03, but also DRB1*14 and to some degree DRB1*15 were *risk factors*, while DRB1*01 and to some extent DRB1*04 instead protected against disease. Interestingly, associations between HLA-DRB1 alleles and disease were in several cases clearly different in the two patient sub-groups; while DRB1*01 strongly protected against disease in non-Löfgren's patients, we found no influence on Löfgren's syndrome. DRB1*03 clearly protected against disease in non-Löfgren's, but in contrast strongly increased the risk for Löfgren's syndrome. DRB1*11 tended to increase the risk for non-Löfgren's disease, but had no such influence on Löfgren's syndrome. The increased risk for sarcoidosis associated with DRB1*14 or DRB1*15 also seemed to be restricted to non-Löfgren's disease. Altogether, these results clearly show the importance of patient phenotyping in order to reveal true genetic associations with sarcoidosis. Comparing data from studies on HLA-associations that have been performed on patient populations including various mixed patient phenotypes may give false results. The need for taking patient phenotype into consideration may in part explain previous heterogeneous results on HLA-associations with sarcoidosis, as may also too small sample sizes.

The discrepant and in some cases opposite genetic associations described here for some of the DRB1 alleles with non-LS and LS patients are in line with the concept that LS is a distinct disease entity, further supported by the finding of a variant of CCR2 that was recently described to associate with LS patients independently of DRB1*03 [15] There were however also several similarities between non-LS and LS patients, especially regarding the influence of DRB1 alleles on the *disease course*. DRB1*03 protected against a non-resolving disease while DRB1*07 weakly increased the risk for a non-resolving disease in both patient sub-groups. Both DRB1*14 and DB1*15 tended to increase the risk for a non-resolving disease in the two subgroups, though with a more pronounced influence of DRB1*14 on non-LS and of DRB1*15 on LS patients. Such similar associations may reflect a capacity to present specific antigens that are linked to a more prolonged disease course, or they may reflect linkages to other genes important for the immune response and for the typical formation of non-caseating granulomas, and to the continuing existence of such granulomas, which in turn could lead to a longer disease course.

Our findings confirm some previous observations on smaller numbers of patients. In a study on three cohorts of sarcoidosis patients from United Kingdom, Poland, and the Czech Republic, DRB1*01 was consistently found to protect against sarcoidosis [21]. Furthermore, DRB1*04 was found to be protective in UK patients only, which was in agreement with previous reports from Sweden [9], Japan [22] and Italy [8]. The protective properties of DRB1*01 and DRB1*04 were suggested to be caused by the presence in these DRB1 alleles of small hydrophobic residues at position 11, which is within a pocket of the antigen-binding groove (pocket 6) and influence the binding of antigenic peptides [21]. Our present study thus confirms the protective effect of especially DRB1*01 in sarcoidosis, however only in non-Löfgren's syndrome patients. Interestingly, DRB1*01 has also been shown to protect against multiple sclerosis (MS), i.e. another immune mediated inflammatory disorder [23].

Several other HLA DRB1 alleles have been shown to be risk factors. In the previously published largest HLA-survey, from the "A Case Control Etiologic Study of Sarcoidosis" (ACCESS) study and including 474 HLA-typed patients, it was reported that especially DRB1*11 but also DRB1*12 and DRB1*15 were risk factors for sarcoidosis [24]. We and others have also previously reported on the increased risk for sarcoidosis associated with DRB1*03, DRB1*14 and DRB1*15 [9,21,25]. Thus, by large a pattern of HLA associations with sarcoidosis is emerging.

In addition, specific HLA alleles can also associate with the disease course. Previous reports have shown especially a strong association between HLA-DRB1*03 and Löfgren's syndrome, and a good prognosis [6-12,14]. Also HLA-DRB1*1501 (strongly linked to DQB1*0602) has previously been associated with a chronic course of sarcoidosis and severe pulmonary sarcoidosis [9,11,12,26].

The reason for HLA class II associations with disease is not clear, but is likely due to their pivotal role for mounting specific immune responses by presenting antigen peptides for T cells, which in sarcoidosis results in lung accumulation and activation of CD4+ T cells, granuloma formation and in some cases chronic inflammation and fibrosis. That specific antigen peptide binding by DRB1 alleles is important in sarcoidosis is supported by the finding of specific HLA class II amino acid epitopes, especially in pocket 4 of the DRB1 molecule, that correlate with disease. Intriguingly, Arg74 in DRB1 pocket 4 was found to independently correlate with radiographic stage I [27]. This particular DRB1 pocket 4 is also important for generating accumulations of T cells expressing the AV2S3 T cell [28], in line with a specific T cell mediated immune response directed against a specific antigenic peptide in the lungs of sarcoidosis patients. Recently, using bioinformatic tools, Saltini et al suggested that HLA-DRB1 alleles of LS patients could recognize significantly higher numbers of bacterial antigen epitopes, especially those derived from mycobacteria [29].

However, the strong linkage disequilibrium (LD) in this region complicates interpretations of HLA associations. It may be that the associations between specific HLA alleles and disease instead reflect associations with other closely linked genes of importance for immune responses, such as tumor necrosis factor (TNF) and lymphotoxin (LT).

For example, DRB1*03 is part of the "8.1 ancestral haplotype", which is associated with a number of other autoimmune diseases, such as myasthenia gravis and systemic lupus erythematosus. This haplotype includes a large number of gene variants that may be important for the immune response, e.g. a defect complement 4 (C4), which could result in a decreased ability to eliminate immune complexes of possible importance for the development of erythema nodosum caused by deposition of immune-complexes [30,31].

In conclusion, this study reveals the importance of sub-grouping sarcoidosis patients when calculating genetic risk factors, since we here found several distinct differences between the patient subgroups and describes new associations between specific HLA-DRB1 alleles and sarcoidosis. In future studies, as we continue and collect more patient data, it will be interesting to study separately subgroups of patients with particular clinical manifestations and specific organ engagements.

## Competing interests

The authors declare that they have no competing interests.

## Authors' contributions

JG designed and coordinated the study, recruited and characterized patients, summarized data and drafted the manuscript. BB performed calculations, intrepreted data and wrote parts of the manuscript. MN, PD and KC helped characterize patients and classify clinical information. JH interpreted clinical information and HLA-data. AE co-designed the study, recruited and characterized patients, intrepreted data and helped writing the manuscript. All authors read and approved the final manuscript.
